# Determinants of healthcare facility utilization for childbirth in *Kuantan Singingi* regency, *Riau* province, Indonesia 2017

**DOI:** 10.1186/s12889-020-09035-3

**Published:** 2020-06-15

**Authors:** Rajunitrigo Sukirman, Tri Yunis Miko Wahyono, Siddharudha Shivalli

**Affiliations:** 1 Department of Epidemiology, Faculty of Public Health, Universitas Indonesia, Prof. Dr. Bahder Djohan Street, Depok, 16424 Indonesia; 2 Department of Disease Prevention and Control, Riau Provincial Health Office, Cut Nyak Dien III Street, Pekanbaru, 28126 Indonesia; 3 Non-Communicable Diseases Regional Technical Advisor, Southeast Asia Regional Office (SEARO), TEPHINET, A Program of the Task Force for Global Health, Inc., Decatur, GA 30030 USA; 4 Department of Medical Statistics, London School of Hygiene & Tropical Medicine, London, WC1E 7HT UK

**Keywords:** Epidemiologic, Determinants, Delivery place, Healthcare facility, Indonesia

## Abstract

**Background:**

Reducing maternal mortality ratio (MMR) is a high priority public health issue in developing countries such as Indonesia. The current MMR in Indonesia is 126/100,000 live births. Optimum use of available healthcare facilities for delivery can avert maternal deaths. This study aimed to determine the factors associated with healthcare facility utilization for childbirth in *Kuantan Singingi* regency, Riau province, Indonesia 2017.

**Methods:**

We conducted a community-based cross-sectional study in 15 sub-districts of *Kuantan Singingi* regency from May–June 2017. We selected 320 mothers from 15 sub-districts who delivered in the last 3 months (February–April 2017). Trained data enumerators collected the relevant data by using a pre-tested semi-structured questionnaire. We used Cox regression analysis to determine the factors associated with delivery at healthcare facilities. Prevalence Ratio (PR) with a 95% confidence interval (CI) for childbirth at healthcare facilities was the key outcome measure.

**Results:**

Only 54.4% (174) of the 320 mothers delivered at healthcare facilities. Knowledge about pregnancy danger signs (PR = 1.59, 95%CI:1.15–2.2), attitude towards healthcare services (PR = 0.79, 95%CI:0.33–1.89), and access to health care services (PR = 0.39, 95%CI:0.18–0.84) were the dominant factors of childbirth at healthcare facilities. There was an interaction between attitude and access to healthcare influencing delivery at healthcare facilities.

**Conclusions:**

Utilization of healthcare facilities for childbirth was low in *Kuantan Singingi* regency. Knowledge of pregnancy danger signs was an independent correlate of childbirth at healthcare facilities. Also, the interaction between attitude and access to healthcare showed a significant influence on childbirth at healthcare facilities. We recommend strengthening of existing maternal and child health program with a particular emphasis on complete and quality antenatal care, health education on danger signs of pregnancy and childbirth, and promoting positive attitudes towards healthcare facilities.

## Background

Reducing Maternal Mortality Ratio (MMR) is the biggest challenge in developing countries. In 2015, the estimated global MMR was 216 per 100,000 live births, with a total of 303,000 maternal deaths [1]. Approximately 99% of the global maternal deaths are reported from developing regions [2]. Sub-Saharan Africa and Southern Asia account for 66 and 22% of the global maternal deaths, respectively [2]. Indonesia, with an MMR of 126 per 100,000 live births (2010–15), is one of 39 countries that are categorized as making progress in reducing maternal deaths [2, 3]. This figure is still far from the MMR target (70 per 1,00,000 live births) of Sustainable Development Goals (SDGs)-2030. Most of the maternal deaths are attributed to complications of childbirth [4]. In addition to maternal mortality, high neonatal mortality is seen in primiparous mothers with complications of childbirth [5]. Complications of childbirth occur due to delays in three phases (delays in seeking healthcare, reaching the healthcare facility, and in receiving adequate care at the point of service). Delivering at a health facility can be achieved by avoiding the first and second phases of these delays [6, 7].

In 2000, the government strengthened its strategy and intervention in reducing MMR through ‘Making Pregnancy Safer’ (MPS). MPS is one of the strategies focusing on the provision and consolidation of maternal health services. MPS strategies are implemented through midwives in villages, midwife partnerships with Traditional Birth Attendants (TBA), and provision of delivery services at all Primary Health Centers (PHCs) [8]. Over the past three decades, Indonesia has made progress in improving maternal health. The proportion of deliveries at healthcare facilities increased from 63% (2007–12) to 70.4% (2010–13) [3, 9].

**Fig. 1:**
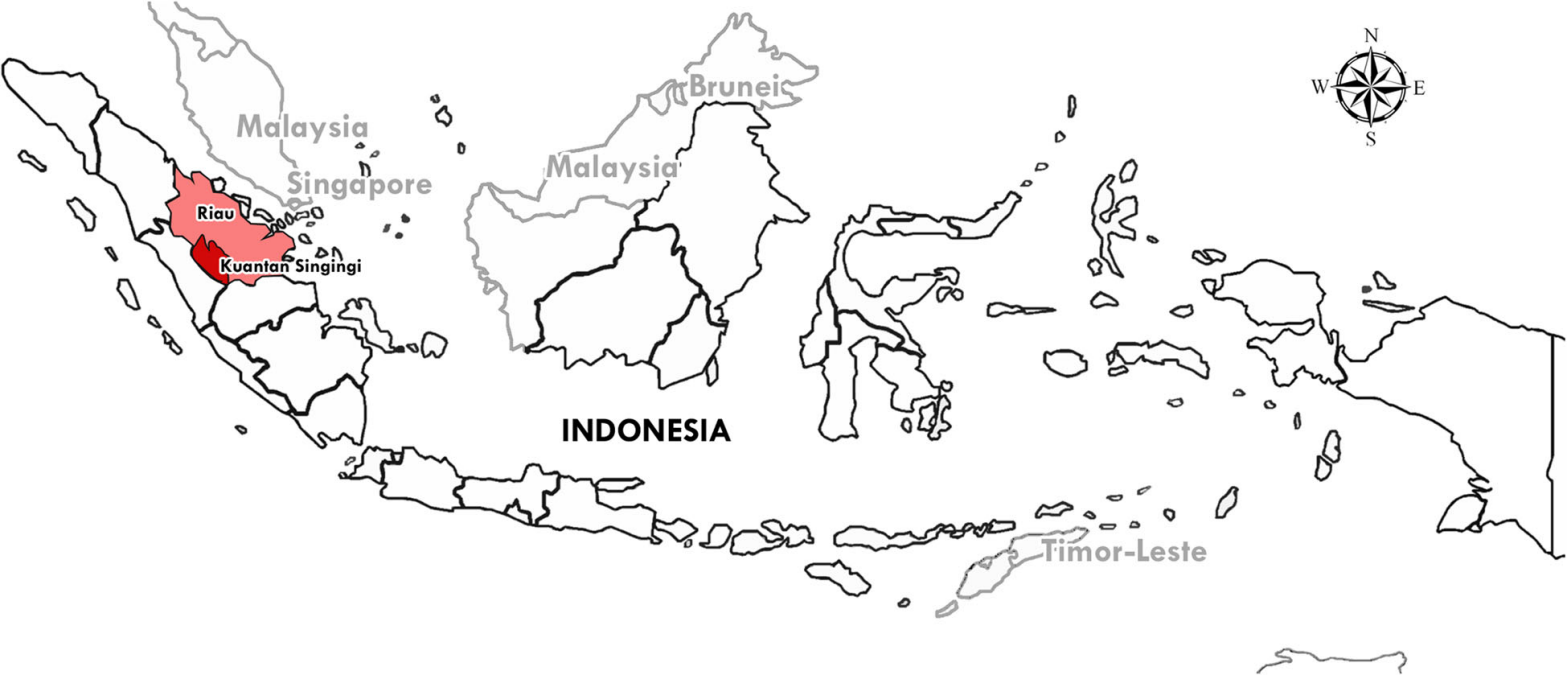
Map of Indonesia showing *Kuantan Singingi* regency, Riau province. We downloaded the Indonesia outline map from FreeWorldMaps (http://www.freeworldmaps.net/asia/indonesia/) in Nov 2019. We obtained written permission to use and adapt it

*Riau* province is located in the central part of Sumatra, Indonesia, with a population of about 6 million [Fig. 1]. *Riau* consists of ten regencies and two autonomous cities. The proportion of deliveries at healthcare facilities in *Riau* province (63%) is lower than the national average [9]. *Kuantan Singingi* regency is located in *Riau* province, which is also experiencing improvement in the maternal health program. Skilled birth attendance and delivery at healthcare facilities in this district in 2016 were 85 and 62%, respectively [10]. However, the proportion of deliveries at health facilities was lower than the Ministry of Health strategic plan target of 81% [10, 11].

Factors influencing the utilization of health services can be divided into predisposing, enabling, and need factors [12]. Predisposing factors are maternal age, occupation, attitudes toward healthcare services, knowledge of danger signs of pregnancy, education, and parity [13–15]. Enabling factors are health insurance, socioeconomic status, access to health care, and frequency of antenatal visits [13–15]. Need factors are perceptions about risks of pregnancy and childbirth, the presence of danger signs of pregnancy and childbirth, and comorbidities in pregnancy [14]. Previous studies have shown an association of above-said factors with the place of delivery [6, 16, 17]. Determining the factors associated with delivery at healthcare facilities would help in fine-tuning of ongoing maternal health interventions in the local setting.

### Objective

To determine the factors associated with healthcare facility utilization for childbirth in *Kuantan Singingi* regency, Riau province, Indonesia 2017.

## Methods

### Study design and setting

We conducted a community-based cross-sectional study in 15 sub-districts of *Kuantan Singingi* regency, Riau province, Indonesia from May–June 2017 [Fig. 1]. *Kuantan Singingi* regency is located in Sumatra island of Indonesia, with a total population of 2.92 million. It consists of 15 sub-districts. The public health infrastructure consists of 25 PHCs (six with Basic Emergency Obstetric and Neonatal Service, known as *PONED*), one hospital with Comprehensive Emergency Obstetric and Neonatal Service is known as *PONEK*, three maternity units, one Mother and Child Hospital and 52 private clinics. *PONED* PHC to population ratio was 0.95 per 100,000 (standard 1:100,000 population), and midwives to population ratio was 112 per 100,000 (standard 100 per 100,000 population).

### Sample size

We estimated the sample size by using the formula for two independent proportions. Based on the reported 50% of the women who delivered at healthcare facilities had high education, an expected response rate of 90%, and a design effect of 1.1, the study required a total sample size of 314 to achieve a power of 80% for detecting a difference in proportions of 17% at a two-sided p-value of 0.05 [14, 18, 19].

### Inclusion criteria

We included all the women aged 15–54 years residing in *Kuantan Singingi* regency and had delivered (irrespective of the place of delivery) in the last 3 months (February–April 2017).

### Sampling

All the mothers who delivered in the last 3 months in 15 sub-districts formed the sampling frame. We obtained a sub-district wise list of mothers who delivered within 3months (between February and April 2017) from village midwives. The list included all the women irrespective of whether she availed any health service and place of delivery. The samples were drawn from each sub-district based on the proportion probability to size [20]. We selected the first woman by simple random sampling. Subsequently, we included all the consecutive enlisted mothers until the required number was achieved for the sub-district.

### Data collection

We conducted this study in collaboration with the heads of PHCs and facilitated by the District health office *Kuantan Singingi.* A semi-structured interview guide was used to collect relevant data. To validate the primary data, we used data from the Mother, and Children Health book or maternal cohort register with village midwife. We trained the data enumerators and supervisors, with a minimum education of bachelor of health, for data collection. Twenty-four data enumerators and six supervisors were trained for 2 days in *Pekanbaru* City, *Riau* province, Indonesia. Training included the following: introducing themselves to participants, explaining the purpose of the study, obtaining the written informed consent, interview technique, filling the responses in the questionnaire, interview practice, and supervision.

## Operational definitions

Healthcare facility for childbirth was defined as delivery at a government hospital, private hospital, PHC, doctor’s practice, midwife practice, or village maternity post [21]. Delivery at village health post or home with a midwife and/or TBA was considered as non-healthcare facility delivery. In Indonesia, a midwife is a woman who has graduated from a midwifery college, registered, and has a license to practice midwifery. TBA is a person who assists a woman during childbirth and acquired skills by conducting deliveries herself or through apprenticeship to other TBA.

Age of the mother was categorized as low risk (20–35 years) and high risk (< 20 years or > 35 years) for childbirth [22]. The highest level of formal education completed by the mother was categorized as basic (no schooling, elementary school, or junior high school) and senior high school/above [23].

Mothers’ occupation was classified as employed (civil servants, military, police, private, self-employed, farmers, and others) and unemployed [24].

We prepared a questionnaire to assess the mothers’ attitudes towards healthcare services. We tested the questionnaire for validity and reliability on 30 participants in *Kampar* regency, Indonesia, whose socio-demographic and cultural profile was similar to the study district. Of the 22 questions, responses to four questions were invalid, and the overall reliability of the questionnaire was high (Cronbach’s alpha: 0.974). Attitude scores were estimated for the degree of tendency to approach, like, expectation or tendency to stay away from, avoid, hate, and dislike antenatal care and delivery at healthcare facilities [25]. Attitude was categorized as positive (score ≥ mean/median) and negative (score < mean/median). Knowledge of pregnancy danger signs (vaginal bleeding, high fever, convulsions, baby in the wrong position, swelling of the feet/hands/face, fainting, difficulty in breathing, and excessive tiredness) was assessed by scoring [9]. It was categorized as good (score ≥ mean/median) and poor (score < mean/median).

Ownership of health insurance was defined as the availability of health protection for mothers to benefit health care and protection in meeting basic health needs provided to those who have paid contributions or fees paid by the government [26]. As described in RISKESDAS, we used eight binomial variables for ownership of valuable goods to construct economic status indices using principal component analysis, tetrachoric and polychoric correlation [9]. Economic status was categorized as high (3rd-5th quintile of the study sample) and low (1st-2nd quintile of the study sample).

Using RISKESDAS questionnaire, we assessed access to health services according to the presence of nearby healthcare facilities, travel time, mode of transport used, transport cost, and ease of transportation [9]. It was categorized as easy (score ≥ mean/median) and difficult (score < mean/median). Antenatal care was considered as complete if the mother had at least four antenatal visits (first within 3 months, second between four and 6 months, twice between seven and 9 months) during pregnancy [27]. Perceived risk of pregnancy and childbirth was assessed according to the respondent's immediate response to the harmful outcomes of pregnancy and childbirth [28]. It was categorized as high (score ≥ mean/median) and low (score < mean/median).

Presence of danger signs of pregnancy (vaginal bleeding, high fever, convulsions, baby in wrong position, swelling of the feet/hands/face, fainting, difficulty in breathing, excessive tiredness) and childbirth (bleeding, convulsions, and or bleeding diagnosis, severe preeclampsia, premature rupture of membranes) were assessed and scored. Mother was categorized as high (score ≥ cut of point Receiver Operating Characteristic, ROC) and low risk (score < cut of point ROC) [27, 29, 30]. Presence of comorbidity in pregnancy, such as malaria, pulmonary tuberculosis, asthma, diabetes mellitus, severe anemia, urinary tract infections, have experienced events that resulted in injury so that daily activities are disrupted were noted [31].

### Statistical analysis

We used STATA**®** version 12 for data analysis. Descriptive statistics were used for categorical and continuous variables [32, 33]. Univariate analysis was done by Chi-square test and prevalence ratio (PR) with 95% confidence interval at a significance limit (α) of 5% [33, 34]. Multivariate analysis was done using a modified Cox Proportional Hazard Regression (cox regression). We used PR in modified cox regression as the prevalence of the outcome was > 10%. In modified cox regression, the timing of the selection of the delivery place was considered as constant or at the same time. Survival time in cox regression was determined by the same number (i.e., 1) with an assumption that the choice of delivery place occurs on the day of the interview. The required PR score for this study was adjusted for Hazard Ratio (HR) resulting from the Cox test since HR was the outcome in the exposed and unexposed groups [35].

All the study variables with *p* ≤ 0.25 on univariate analysis were considered into a multivariable model. The possible effects of modification/interaction of variables into the model were examined. The modified/interaction effect assessment should have a meaningful p-value with the important interaction variable to be included in the model. We assessed the interaction by entering one by one variable that was suspected of having interaction in the initial model. An initial Hierarchically Well Formulated Model (HWF Model) or most complete model was created. In the HWF model (full model), variable having *p* value> 0.05 was eliminated, starting from the largest p-value. The result of this modeling is a reduced model. Based on the Log LR (Likelihood Ratio) test, the variable was considered not significant and not included in the model if the LR > 3.8. The final model was described based on the final test results and interaction of dependent and independent variables.

### Ethical approval

Ethical Commissions of Research and Community Service, Faculty of Public Health, University of Indonesia, Indonesia approved the study (275/UN2.F10/PPM.00.02/2017). Trained data enumerators obtained the written informed consent from the study participants for voluntary participation. Informed consent was taken from the parent or legal guardian if the woman aged < 18 years. Investigators followed the ethical principles of Helsinki Declaration-2013 and International Ethical Guidelines for Epidemiological Studies [36, 37].

## Results

We approached 320 eligible mothers, and all of them participated in the study. Their mean age (±SD) was 28 (±5.75) years. Table 1 shows the mothers’ key socio-demographic and obstetric characteristics. Of the 320 mothers, 174 (54.38, 95%CI: 48.8–59.9%) delivered at healthcare facilities. Private hospitals (77, 24.1%) and midwife practice (57, 17.8%) were the preferred places of delivery. Nearly two-thirds (63% and overall 28.8%) of the non-healthcare deliveries were conducted at home by midwife [Table 2].

**Table 1:** Key socio-demographic and obstetric characteristics of mothers in *Kuantan Singingi* regency, Riau province, Indonesia 2017 (*N* = 320)

Variable	n	%
**Mother’s age**		
Low risk (20–35 years)	253	79.1
High risk (< 20 or > 35 years)	67	20.9
**Education**		
Senior high school/above	194	60.6
Basic	126	39.4
**Employment status**		
Employed	68	21.3
Unemployed	252	78.8
**Parity**		
1–2	229	71.6
≥ 3	91	28.4
**Economic status**		
High	155	48.4
Low	165	51.6
**Place of delivery**		
Health facility	174	54.4
Non-health facility	146	45.6
**Attitudes towards health services**		
Positive	186	58.1
Negative	134	41.9
**Ownership of health insurance**		
Insured	156	48.8
Not insured	164	51.3
**Knowledge of danger signs of pregnancy**		
Good	191	59.7
Poor	129	40.3
**Antenatal care visits**		
Complete (≥4 visits)	234	73.1
Incomplete (< 4 visits)	86	26.9
**Access to health services**		
Easy	291	90.9
Difficult	29	9.1
**Perceived risk of pregnancy and childbirth**		
Good	175	54.7
Bad	145	45.3
**Presence of danger sign of pregnancy**		
High risk	7	2.2
Low risk	313	97.8
**Presence of danger sign of childbirth**		
High risk	36	11.3
Low risk	284	88.8
**Comorbidities in pregnancy**		
Yes	16	5
No	304	95

**Table 2:** Distribution of recently delivered mothers according to place of childbirth in *Kuantan Singingi* regency, Riau province, Indonesia 2017 (*N* = 320)

Place of childirth	n	%
Goverment hospital	7	2.2
Private Hospital	77	24.1
Primary healthcare centre (*Puskesmas*)	7	2.2
Doctor’s Practice	21	6.6
Midwife Practice	57	17.8
Village Maternity Post	5	1.6
Village Health Post	9	2.8
Home by Midwife	92	28.8
Home by Doctor	4	1.3
Home by TBA (*Dukun beranak*)	13	4.1
House by Midwife and TBA	27	8.4
Home by Doctor partner, Midwife and TBA	1	0.3

Table 3 shows the association of various study variables with childbirth at healthcare facilities. The following factors had a significant association (*p* < 0.05) with childbirth at healthcare facilities: maternal education, maternal employment, attitudes towards healthcare services, knowledge of danger signs of pregnancy, complete antenatal care visits, access to health care, and perceived risk of pregnancy, and childbirth.

**Table 3:** Association of various study variables with child birth at healthcare facilities in *Kuantan Singingi* regency, Riau province, Indonesia 2017 (*N* = 320)

Variable	Place of delivery				PR	PR (95% CI)
	Healthcare facility (***n*** = 174)		Non-healthcare facility (***n*** = 146)			
	n	%	n	%		
**Predisposing factors**						
**Mother’s Age**						
Low risk (20–35 years)	135	53	118	47	0.91	0.72–1.15
High risk (< 20 or > 35 years)	39	58	28	42	1	
**Mother’s education**						
Senior high school/above	116	59.8	78	40.2	1.29*	1.04–1.62
Basic	58	46	68	54	1	
**Employment status**						
Employed	45	66	23	34	1.29*	1.04–1.59
Unemployed	129	51	123	49	1	
**Parity**						
1–2	126	55	103	45	1.04	0.83–1.31
≥ 3	48	53	43	47	1	
**Attitudes towards health services**						
Positive	124	66.7	62	33.3	1.78*	1.4–2.27
Negative	50	37.3	84	62.7	1	
**Knowledge of danger signs of pregnancy**						
Good	124	64.9	67	35.1	1.55*	1.27–1.89
Poor	50	38.8	79	61.2	1	
**Enabling factors**						
**Economic status**						
High	80	46	75	51	0.89	0.71–1.12
Low	94	54	71	49	1	
**Ownership of health insurance**						
Insured	90	58	66	42	1.12	0.92–1.37
Not insured	84	51	80	49	1	
**Antenatal care visits**						
Complete (≥4 visits)	141	60	93	40	1.57*	1.17–2.09
Incomplete (< 4 visits)	33	38	53	62	1	
**Access to health services**						
Easy	152	52.2	139	47.8	0.68*	0.54–0.86
Difficult	22	75.9	7	24.1	1	
**Need factors**						
**Perceived risk of pregnancy and childbirth**						
Good	108	61.7	67	38.3	1.35*	1.09–1.67
Bad	66	45.5	79	54.5	1	
**Presence of danger signs of pregnancy**						
High risk	4	57.1	3	42.9	1.05	0.54–2.01
Low risk	170	54.3	143	45.7	1	
**Presence of danger signs of childbirth**						
High risk	20	55.6	16	44.4	1.02	0.75–1.39
Low risk	154	54.2	130	45.8	1	
**Comorbidity in pregnancy**						
Present	13	81	3	19	1.53*	1.18–1.98
Absent	161	53	135	47	1	

*p **< 0.05**

In multivariate analysis, an initial model was prepared with all the variables with *p* < 0.25 on bivariate analysis, and interaction assessment was done by forward entry. The following nine variables were included in the initial model: maternal education, maternal employment, attitudes towards healthcare services, knowledge of danger signs of pregnancy, health insurance, complete antenatal care visits, access to health care, and perceived risk of pregnancy and childbirth.

Table 4 shows the final model by backward elimination for determinants of childbirth at healthcare facilities. Good knowledge of danger signs of pregnancy (PR:1.59, 95%CI:1.15–2.2) and access to healthcare services (PR:0.39, 95%CI:0.18–0.84) were the determinants of childbirth at healthcare facilities in *Kuantan Singingi* regency, Indonesia.

**Table 4:** Final cox regression model of determinants of childbirth at health facilities in *Kuantan Singingi* regency, Riau province, Indonesia 2017 (*N* = 320)

Variable	Coef β	SE	PR	95% CI	***p-value***
Knowledge of danger signs of pregnancy	0.469	0.268	1.59	1.15–2.22	0.005*
Access to health services	−0.922	0.153	0.39	0.18–0.84	0.017*
Attitudes towards health services	−0.231	0.351	0.79	0.33–1.89	0.601
Attitude * access to health services	0.880	1.155	2.41	0.94–6.16	0.066

*p < 0.05

In this research, there was an interaction between attitude and access to healthcare services, so its PR value cannot be interpreted directly, but through some calculation phases [Table 5]. Based on the calculation of interaction PR, mothers who had easy access to healthcare were more likely to choose healthcare facilities for childbirth irrespective of their attitudes (positive/negative) towards healthcare facilities when compared to mothers who had a negative attitude and difficult access (reference group). When compared to the reference group, mothers with difficult access to the healthcare facility and positive attitude towards healthcare facilities were more likely to deliver at the non-healthcare facility.

**Table 5:** Results of prevalence ratio interaction with health service attitudes and access to health services among mothers in *Kuantan Singingi* regency, Riau province, Indonesia 2017 (*N* = 320)

Interaction variable	PR interaction	95% CI
Positive attitude, easy access	1.91	1.34–2.73
Positive attitude, difficult access	0.793	0.33–1.89
Negative attitude, easy access	2.41	0.94–6.17
Negative attitude, difficult access	1 (reference)	–

Attitude (1 = positive attitude, 0 = negative attitude), Access (1 = easy access, 0 = difficult access)

## Discussion

In *Kuantan Singingi* regency, the utilization of healthcare facilities for childbirth was much lower than the Ministry of Health’s strategic plan target of 81% [10, 11]. A qualitative research in *Tangerang* district, *Banten*, Indonesia, reported that mothers were more comfortable to deliver at home in the presence of family members, who provide support during delivery. Mothers choose TBA as a birth attendant due to customary use of TBA services [38]. Similar qualitative studies in *Kuantan Singingi* regency could help to understand the reasons for preference for home deliveries.

Among the deliveries at healthcare facilities, every fourth and sixth woman delivered at private hospitals and midwife practice, respectively. Previous studies in Indonesia reported that the type of healthcare facility was chosen based on the proximity, infrastructure, skilled personnel, previous experience of parents, and in-laws [28, 39]. Similar factors may have played a role in deciding the type of health facility for delivery in this study.

In Indonesia, TBA (known as ‘*Dukun beranak’*) is also very popular for conducting deliveries. However, these do not have any medical expertise, and their role is restricted (by the Ministry of Health) to support the mothers. A midwife or medical personnel should conduct the delivery. Ministry of Health developed a partnership program between TBAs and midwives so that TBAs act as a link between the healthcare system and community [40]. TBA is expected to mentor pregnant women for full antenatal care, delivery at healthcare facility, escort her to midwife for delivery and care of the newborn. In the study setting, there is a need for re-orientation and capacity building of midwives and TBAs to enhance childbirth at healthcare facilities.

Various studies across the globe have studied the association between childbirth at healthcare facilities and maternal factors such as age, education status, employment status, and parity [7, 13, 41–45]. In this study, none of these showed significant association with childbirth at healthcare facilities.

Previous studies from Indonesia and Ghana have reported the positive impact of health insurance on the utilization of healthcare facilities for delivery [13, 46]. In 2014, Indonesia launched a national health insurance scheme (*Jaminan Kesehatan Nasional*, JKN). It aims to provide universal health coverage to the entire Indonesian population by 2019. In this study, almost half of the mothers had health insurance. However, there was no association between health insurance and the utilization of healthcare facilities for childbirth. Whether it was a serendipitous finding or issues such as out-of-pocket expenditure, poor implementation, or acceptability of JKN, etc. needs further exploration.

Lower utilization of existing healthcare facilities by economically poor mothers has been consistent in previous studies from Indonesia, Thailand, other Asian countries, Africa, and Latin America [13, 47–49]. However, this was not significant in this study, although 51.6% of the mothers were from a low economic background. This could be attributed to improved use of healthcare services by the poor in the regency or methodological differences in assessing the economic status in different countries.

Studies from Indonesia [15] and Ethiopia [46] have shown that mothers with positive attitudes towards available healthcare services are more likely to deliver at a healthcare facility. However, we did not find a similar significant association in this study.

In this study, mothers with easy access to healthcare services were less likely to use healthcare facilities for childbirth when compared to their counterparts. The observed interaction between access to healthcare and attitudes is a possible explanation. Irrespective of their attitudes towards healthcare facilities, mothers with easy access to healthcare facility were more likely to choose healthcare facility for childbirth. However, other factors such as cost of healthcare service (especially in urban areas), cultural factors, and service time influencing childbirth at healthcare facilities were not studied in this study [13, 38].

According to Fosu GB [50], the utilization of healthcare services depends on the perceived risk of present condition and benefits of the treatment. Similar findings were also seen in this and another study from Indonesia [15]. In this study, knowledge of danger signs of pregnancy was an independent correlate of childbirth at healthcare facilities. Perceived risk plays a role in shaping mothers’ decisions and actions and influenced by other factors such as antenatal care visits, age, occupation, etc. [7]. Women with sufficient knowledge of danger signs are capable of early recognition of potentially life-threatening complications and avert unnecessary delay in seeking healthcare [51]. Hence, education about the danger signs during antenatal care visits should be emphasized. However, the presence of danger signs of pregnancy and childbirth were not associated with delivery at healthcare facilities, although others reported contrary findings [49, 50, 52]. Very few mothers experienced danger signs of pregnancy (2.2%) and childbirth (11.3%), differences in the study setting could be a possible explanation. Mothers with comorbidities during pregnancy are at high risk of morbidity and mortality, and they directly affect the utilization of healthcare services [12, 53]. However, in this study, we did not observe a significant association between comrbidity and childbirth at healthcare facilities.

In a developing country like Indonesia, especially in the Muslim culture, the decision-making system is patriarchal. Family members like husband, parents, and, in-laws play a crucial role in deciding the place of childbirth [38, 54, 55]. However, the present study focused only on the woman as a participant.

## Strengths and limitations

The study participants were selected from 15 sub-districts and fairly represented recently delivered mothers in the regency. To minimize the recall bias, only mothers delivered in the last 3 months were included, and, wherever possible, information obtained from the interviews was verified with Mother, and Children Health book records. To minimize the possibility of misclassification bias, we used RISKESDAS standard questionnaire (2010 and 2013) to assess the economic status and access to health services. RISKESDAS economic status questionnaire is relatively easy and widely used in developing countries. The limitation is determining the weight for each item. The access to health services questionnaire uses weighted difficulty levels. However, the respondent’s answer might be subjective, such as determining travel time. Associations observed in this study may not imply causality owing to the cross-sectional study design. This study did not consider husband, parents, and, in-laws-related factors which may influence in deciding the place of childbirth.

## Conclusions

In *Kuantan Singingi* regency, childbirth at healthcare facilities was low. We recommend strengthening of existing maternal and child health program with a particular emphasis on imparting complete and quality antenatal care, health education on danger signs of pregnancy and childbirth, and promoting positive attitudes towards healthcare facilities. These interventions are known to change the behavior and reduce the maternal and neonatal mortalities in low-resources settings [56, 57]. Further studies are needed to explore the role of health insurance and accessibility to healthcare in utilization of available facilities.

## Data Availability

The datasets used and/or analyzed during the current study are available from RS (rigoners@yahoo.co.id) on reasonable request.

## Abbreviations

CI: Confidence Interval
HR: Hazard Ratio
HWF Model: Hierarchically Well Formulated Model
*JKN*: *Jaminan Kesehatan Nasional* (National Health Insurance)
LR: Likelihood Ratio
MMR: Maternal Mortality Ratio
MPS: Making Pregnancy Safer
PHC: Primary Health Centers
*PONED*: Basic Emergency Obstetric and Neonatal Service
*PONEK*: Comprehensive Emergency Obstetric and Neonatal Service
PR: Prevalence Ratio
RISKESDAS: Indonesia Basic Health Research
ROC: Receiver Operating Characteristic
SDGs: Sustainable Development Goals
TBA: Traditional Birth Attendants

## References

[1] Alkema L, Chou D, Hogan D, Zhang S, Moller A (2016). Global, regional, and national levels and trends in maternal mortality between 1990 and 2015, with scenario-based projections to 2030: A systematic analysis by the UN maternal mortality estimation inter-agency group. Lancet.

[2] World Health Organization. Trends in maternal mortality: 1990 to 2015. Estimates by WHO, UNICEF, UNFPAM, World Bank Group and the United Nations Population Division: World Health Organization; 2015. Available from http://www.who.int/reproductivehealth/publications/monitoring/maternal-mortality-2015/en/.

[3] Nuraini S, Wahyuni T, Windiarto E, Karyono OS (2016). Profil Penduduk Indonesia Hasil SUPAS 2015.

[4] Chowdhury ME, Ahmed A, Kalim N, Koblinsky M (2009). Causes of maternal mortality decline in matlab, Bangladesh. J Health Popul Nutr.

[5] Prameswari MF (2007). Kematian perinatal di Indonesia dan faktor yang berhubungan, tahun 1997-2003. Jurnal Kesehatan Masyarakat Nasional.

[6] Thaddeus S, Maine D (1994). Too far to walk: maternal mortality in context. Soc Sci Med.

[7] Stekelenburg J, Kyanamina S, Mukelabai M, Wolffers I, van Roosmalen J (2004). Waiting too long: Low use of maternal health services in Kalbo, Zambia. A Eur J TMIH.

[8] Departemen Kesehatan RI (2005). Rencana strategis nasional Making Pregnancy Safer (MPS) di Indonesia 2001–2010.

[9] Badan Penelitian dan Pengembangan Kesehatan (2013). Riset Kesehatan Dasar, Riskesdas.

[10] Dinas Kesehatan Kabupaten Kuantan Singingi (2016). Profil Kesehatan Kabupaten Kuantan Singingi.

[11] Dinas Kesehatan Kabupaten Kuantan Singingi (2017). Laporan PWS KIA Kabupaten Kuantan Singingi tahun 2016.

[12] Andersen R, Newman J (2005). Societal and individual determinants of medical care utilization in The United State. Milbank Q.

[13] Arief M S. Determinan pemilihan persalinan di fasilitas kesehatan (analisis data riset kesehatan dasar tahun 2010). Jurnal Kesehatan Reproduksi. 2014;5(3) [cited 2017 June 14]Available from: http://ejournal.litbang.kemkes.go.id/index.php/kespro/article/ view/3892/3737.

[14] Maimunah (2010). Determinan Pemanfaatan Layanan Persalinan (Analisis Data SDKI 2007). [Tesis].

[15] Khaeruddin (2012). Determinan pemanfaatan pertolongan persalinan oleh tenaga kesehatan di Puskesmas Cijeruk tahun.

[16] Stephenson R, Baschieri A, Clements S, Hennink M, Madise N. Contextual influences on the use of health facilities for Childbirth in Africa. AJPH. 2006;96(1). 10.2105/AJPH.2004 January [cited 2017 April 12]. Available from: . 57422. 10.2105/AJPH.2004.057422 PMC1470452 16317204

[17] Bonfrer I, Breebaart L, Van de Poel E. The effects of Ghana’s national health insurance scheme on maternal and infant health care utilization. PLoS One. 2016;11(11) November [cited 2017 April 12]. Available from: http://journals.plos.org/plosone/article?id=10.1371/journal.pone.0165623. 10.1371/journal.pone.0165623 PMC5106190 27835639

[18] Sabri L, Hastono SP (2014). Statistik Kesehatan.

[19] Lemeshow S (1997). Besar sampel dalam penelitian kesehatan.

[20] Sastroasmoro S, Ismael S (2011). Dasar-dasar penelitian: metodologi penelitian klinis.

[21] Mubarak WI (2014). Ilmu kesehatan masyarakat: konsep dan aplikasi dalam kebidanan.

[22] Indonesia KKR (2010). Pedoman Pemantauan Wilayah Setempat Kesehatan Ibu dan Anak (PWS KIA).

[23] Undang-Undang Nomor 20 Tahun 2003- Sistem Pendidikan Nasional. Produk Hukum | JDIH Kementerian Sekretariat Negara. 2003. [cited 8 January 2020]. Available from: https://jdih.setneg.go.id/viewpdfperaturan/P01199/UU0202003.

[24] Nurasih RD (2015). Faktor-faktor yang berhubungan dengan persalinan pada non fasilitas kesehatan di Indonesia tahun 2013.

[25] Purwanto H (1998). Pengantar perilaku manusia untuk keperawatan.

[26] Kementerian Kesehatan RI (2013). Peraturan Menteri Kesehatan Republik Indonesia Nomor 71 tahun 2013 tentang pelayanan kesehatan pada jaminan kesehatan nasional.

[27] Indonesia KKR (2015). Buku Kesehatan Ibu dan Anak.

[28] Suryawati C. Faktor Sosial Budaya dalam Praktik Perawatan Kehamilan, Persalinan, dan Pasca Persalinan (Studi di Kecamatan Bangsri Kabupaten Jepara). Jurnal Promosi Kesehatan Indonesia. 2007;2(1) January [cited 2017 April 12];Available from: http://www.ejournal.undip.ac.id/index.php/jpki/article/view/2800.

[29] Rochjati P. Skrining Antenatal Pada Ibu Hamil. 2nd ed. Surabaya: Airlangga University Press; 2011.

[30] Badan Penelitian dan Pengembangan Kesehatan (2010). Riset Kesehatan Dasar, Riskesdas 2010.

[31] Reeder SJ, Martin LL, Koniak-Griffin D. Keperawatan maternitas: kesehatan wanita, bayi dan keluarga. vol. 2. 18th ed. Jakarta: EGC; 2014.

[32] Dahlan MS (2011). Statistik untuk kedokteran dan kesehatan.

[33] Hastono SP (2007). Analisis data kesehatan.

[34] Gerstman B. Epidemiology kept simple: an introduction to traditional and modern epidemiology. 3rd ed. Oxford: Wiley-Blackwell; 2013.

[35] Kleinbaum DG, Klein M (2010). Logistic regression: a self-learning text.

[36] World Medical Association (2001). World medical association declaration of Helsinki: Ethical principles for medical research involving human subjects. Bull World Health Organ.

[37] Council for International Organization of Medical Science (2008). International ethical guidelines for epidemiological studies.

[38] Eryando T. Alasan pemeriksaan kehamilan dan pemilihan penolong persalinan. Jurnal Administrasi Kebijakan Kesehatan. 2006:47–51 [cited 2017 April 12]. Available from http://journal.unair.ac.id/download-fullpapers-8.Tris%20Eryando.pdf.

[39] Susilowati R (2001). Pola pengambilan keputusan keluarga dan penolong persalinan dalam memutuskan merujuk ibu bersalin ke Rumah Sakit pada kasus kematian ibu bersalin di Kabupaten Semarang tahun 2000.

[40] Direktorat Jenderal Bina Kesehatan Masyarakat (2008). Pedoman kemitraan bidan dan dukun.

[41] Burgard S (2004). Race and pregnancy-related care in Brazil and South Africa. Soc Sci Med J.

[42] Raghupathy S (1996). Education and the use of maternal health care in Thailand. Soc Sci Med J.

[43] Thorsen VA, Sundby J, Malata A (2012). Piecing together the maternal death puzzle through narratives: the three delays model revisited. J PLoS ONE.

[44] Gabrysch S, Campbell OMR (2009). Still too far to walk: literature review of the determinants of delivery service use. BMC Pregnancy Childbirth.

[45] Jekti RP, Mutiatikum D. Hubungan antara kepatuhan antenatal care dengan pemilihan penolong persalinan. Jurnal Kesehatan Reproduksi. 2011;1(2) April [cited 2017 April 12]. Available from: http://ejournal.litbang.depkes.go.id/index.php/kespro/article/view/1348.

[46] Debelew GT, Afewok MF, Yalew AW. Factors affecting birth preparedness and complication readiness in Jimma Zone, South West Ethiopia: a multilevel analysis. Pan Afr Med J. 2014;19(272) November [cited 2017 April 12]. Available from: https://www.ncbi.nlm.nih.gov/pmc/articles/PMC4391899/pdf/PAMJ-19-272.pdf. 10.11604/pamj.2014.19.272.4244 PMC4391899 25870727

[47] Langi FLFG, Langi G. Barriers to delivery care by skilled attendants in Sulawesi Utara. J Public Health Development. 2009;7(3) Sept-Dec [cited 2017 April 12]; Available from: http://www.aihd.mahidol.ac.th/sites/default/files/images/new/pdf/journal/sepdec2009/1–13.pdf.

[48] Pomeroy A, Koblinsky M, Alva S. Private delivery care in developing countries: trends and determinants. Demographic Health Res. 2010; October [cited 2017 April 12]; No. 76. Available from: http://dhsprogram.com/pubs/pdf/WP76/WP76.pdf.

[49] Chackraborty N, Islam MA, Chowdhury RI, Bari W, Akhter HN (2003). Determinants of the use of maternal health services in rural Bangladesh. Health Promot Int.

[50] Fosu GB (1994). Childhood morbidity and health services utilization: cross-national comparisons of user-related factors from DHS data. Soc Sci Med.

[51] JHPIEGO (2004). Maternal and neonatal health. Monitoring birth preparedness and complication readiness, tools and indicators for maternal and newborn health. Johns Hopkins, Bloomberg school of Public Health, Center for communication programs, Family Care International.

[52] Mahwati Y (2013). Pemanfaatan pelayanan kesehatan ibu di Jawa Barat. Jurnal Kesehatan Masyarakat Nasional.

[53] Sibai BM (2005). Diagnosis, prevention, and management of eclampsia. Obstetric Gynocol.

[54] Imran LOA, Asfian P (2016). Rahmatia. Determinants of Mother’s choice of place delivery in Community of Bajo, Muna District: A qualitative study. Public Health Indonesia.

[55] Efendi F, Ni'mah AR, Hadisuyatmana S, Kuswanto H, Lindayani L, Berliana SM (2019). Determinants of facility-based childbirth in Indonesia. ScientificWorldJournal..

[56] Koblinsky M, Favin M, Kureshy N, Elder L. Behavioral Dimensions of Health and Survival. Mother Care Matters. 2000;9(3):1–19.

[57] Soubeiga D, Gauvin L, Hatem MA, Johri M (2014). Birth Preparedness and Complication Readiness (BPCR) interventions to reduce maternal and neonatal mortality in developing countries: systematic review and meta-analysis. BMC Pregnancy Childbirth.

